# Straightforward synthesis of quinazolin-4(3*H*)-ones *via* visible light-induced condensation cyclization†

**DOI:** 10.1039/d1ra07944a

**Published:** 2022-01-07

**Authors:** Wuji Sun, Xue Ma, Yuqi Pang, Lifeng Zhao, Qidi Zhong, Chunyan Liu, Qiangwen Fan

**Affiliations:** School of Pharmacy, North China University of Science and Technology Tangshan 063210 China chunyanliu@ncst.edu.cn; School of Chemistry, Biology and Materials Science, East China University of Technology Nanchang 330013 China fanqw2019@ecut.edu.cn

## Abstract

A green, simple and efficient method is developed for the synthesis of quinazolin-4(3*H*)-ones *via* visible light-induced condensation cyclization of 2-aminobenzamides and aldehydes under visible light irradiation. The reaction proceeds using fluorescein as a photocatalyst in the presence of TBHP without the need for a metal catalyst. In addition, this reaction tolerates a broad scope of substrates and could afford a variety of desirable products in good to excellent yields. Thus, the present synthetic method provides a straightforward strategy for the synthesis of quinazolin-4(3*H*)-ones.

## Abstract 2

Visible light was used as a readily available and renewable clean energy source for the green and metal catalyst free synthesis of quinazolin-4(3*H*)-ones. High and excellent yields of the desired products were obtained with good functional group tolerance.

## Graphical abstract

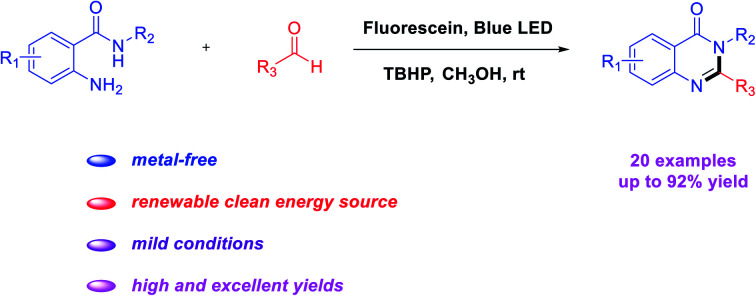

In recent years, synthesis of nitrogen-containing heterocycles has drawn considerable attention due to their widespread occurrence in natural and synthetic organic molecules.^1^ Among them, quinazolin-4(3*H*)-ones are common core structures found in a large number of natural products and synthetic drugs showing a broad range of biological and therapeutic activities (Fig. 1). For example, Pegamine, isolated from *Peganum harmala*, exhibits cytotoxic activity.^2^ Afloqualone is a centrally acting muscle relaxant useful in the management of various conditions, including cerebral palsy, cervical spondylosis, and multiple sclerosis.^3^ Bouchardatine could significantly reduce lipid accumulation, and mainly inhibited early differentiation of adipocytes through proliferation inhibition and cell cycle arrest in a dose-dependent manner.^4^ Idelalisib have been shown to exhibit a broad spectrum of antimicrobial, antitumor, antifungal and cytotoxic activities.^5^ Ispinesib is one of the most potent kinesin spindle protein (KSP) inhibitors and is currently in clinical trials for cancer treatment.^6^ Sclerotigenin, isolated from organic extracts of the sclerotia of penicillium sclerotigenum, is responsible for most of the antiinsectan activity against crop pests.^7^ Besides, a number of quinazolin-4(3*H*)-ones have been synthesized to provide synthetic drugs and to design more effective medicines.^8,9^

**Fig. 1:**
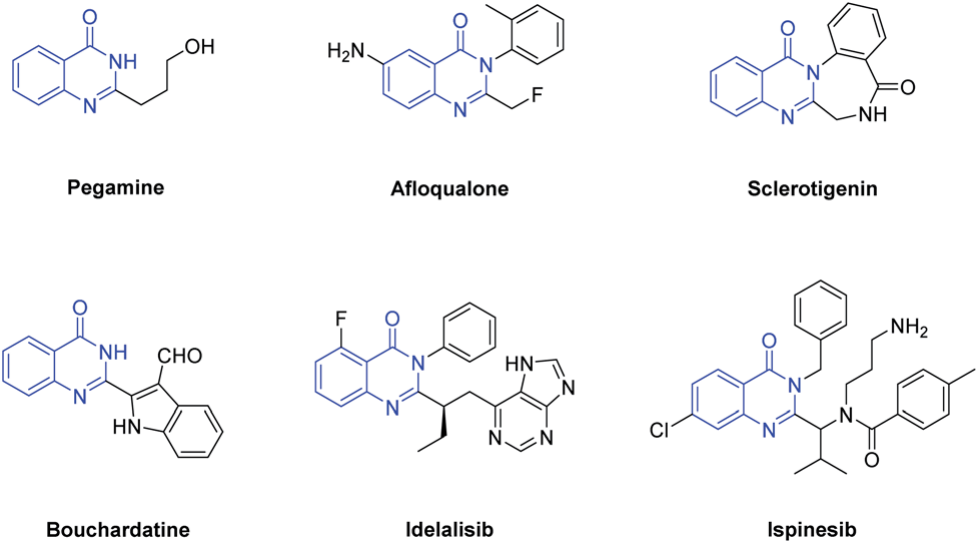
Representing natural and synthetic molecules containing quinazolin-4(3*H*)-one moieties.

Owing to their pharmacological importance, considerable attention has been devoted to the development of simple and efficient methods for their construction (Scheme 1). Typically, quinazolin-4(3*H*)-ones are synthesized by acid or base-catalyzed condensation of amides with alcohols/aldehydes.^10,11^ In some cases, an excess of hazardous oxidants, for instance KMnO_4_, I_2_, and 2,3-dichloro-5,6-dicyano-1,4-benzoquinone (DDQ), are employed in the reaction, which are significant limitations in this method.^12–14^ Over the past decades, various metal-based catalysts such as copper-catalyzed cyclization of 2-halobenzoic acids with amidines, palladium-catalyzed benzylic C–H amidation with benzyl alcohols and vanadium-catalyzed redox condensation of benzamides with alcohols or aldehydes have been reported for the synthesis of quinazolin-4(3*H*)-ones.^15–17^ Although these approaches result in an excellent formation of the product, most of them are suffering from its own limitations such as corrosive or non-benign acid/base catalyst, hazardous oxidants, precious metal-based catalysts, complexity in work-up and relatively harsher reaction conditions. Therefore, development of a green, simple and efficient synthetic approach for the preparation of quinazolin-4(3*H*)-ones from inexpensive and easily available starting materials under relatively mild conditions is desirable.

**Scheme 1:**
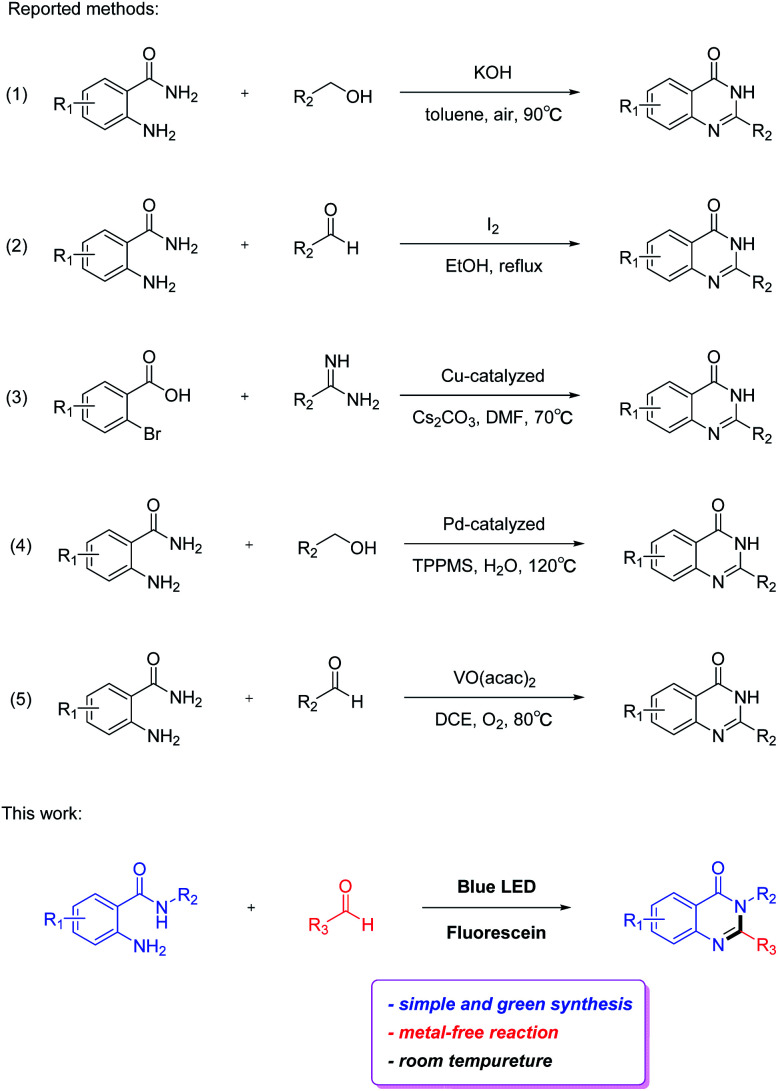
Our work for synthesis of quinazolin-4(3*H*)-ones.

Visible light-induced reaction, which is widely recognized as an attractive “green synthesis pathway” in organic synthesis, has become a fast-developing research area in the past decades.^18–21^ In spite of simple operation and mild reaction conditions, the common transition metals (*e.g.*, iridium and ruthenium) employed as photocatalysts are usually expensive, toxic and not easily available. Nevertheless, organic dyes, which have shown similar photocatalytic activity in some reactions, could be more attractive candidates than the common transition metals, because they are usually relatively cheap, less toxic and accessible.^22^ In particular, fluorescein has been very recently studied as photocatalyst due to its cheap and commercially available characteristics.^23^

In this study, we focus our attention on developing a straightforward method to prepare quinazolin-4(3*H*)-ones with 2-aminobenzamides and aldehydes using fluorescein as photocatalyst *via* visible light-induced condensation cyclization, in which the synthesis of quinazolin-4(3*H*)-ones would proceed in good to excellent yields under mild conditions without the need for metal catalyst (Scheme 1). This method lays a solid foundation for the synthesis of quinazolin-4(3*H*)-ones. Moreover, this visible light-induced strategy has great potential in the synthesis of other types of useful organic molecules.

At the initial stage of investigation, 2-aminobenzamide (1a) and benzaldehyde (2a) were chosen as the model substrates under blue LED irradiation, and a series of reaction conditions including photocatalysts, oxidants, solvents and reaction time were optimized. Initially, the reaction was performed in the presence of 10 mol% fluorescein as photocatalyst and the desired product, 2-phenylquinazolin-4(3*H*)-one (3aa), was obtained in 89% yield (Table 1, entry 1). In a different series, the use of rhodamine B or eosin Y led to a decreasing yield of 3aa (Table 1, entries 2 and 3). When the amount of fluorescein was improved to 15 mol%, the same yield of 3aa was obtained compared to using 10 mol% fluorescein (Table 1, entry 4). However, the lower yield was obtained when the reaction was carried out with 5 mol% fluorescein (Table 1, entry 5). Subsequently, the influence of oxidants on the yield of the product was studied. Of the examined oxidants, TBHP showed the best performance giving 3aa in the highest yield, whereas the oxidative efficiency other oxidants such as K_2_S_2_O_8_, DDQ and O_2_ were all inferior to TBPB (Table 1, entries 6–8). Then, various solvents were screened and it was found that 3aa could be obtained in 89% yield in CH_3_OH, while a reduction in yield was observed upon performing the reaction employing other solvents such as THF, DMF, PhMe and CH_3_CN (Table 1, entries 9–12). Finally, we studied the effect of the reaction time on the reaction and found that 3 hours was kept as the optimum reaction time (Table 1, entries 1, 13 and 14). In summary, it was determined that the optimal reaction conditions were 2-aminobenzamide (1.0 mmol) and benzaldehyde (1.5 mmol) as the reaction substrates, fluorescein (10 mol%) as the photocatalyst, TBHP (2.0 mmol) as the oxidant, CH_3_OH (20 mL) as the solvent and 30 W blue LED as the light source at room temperature for 3 hours.

**Table 1:** Optimization of the reaction conditions^a^

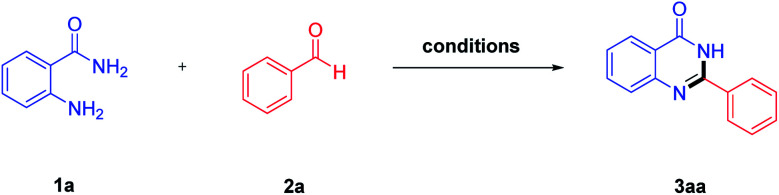					
Entry	Photocatalyst (mol%)	Oxidant	Solvent	Time (h)	Yield^b^ (%)
1	Fluorescein (10 mol%)	TBHP	CH_3_OH	3	89
2	Rhodamine B (10 mol%)	TBHP	CH_3_OH	3	54
3	Eosin Y (10 mol%)	TBHP	CH_3_OH	3	62
4	Fluorescein (15 mol%)	TBHP	CH_3_OH	3	89
5	Fluorescein (5 mol%)	TBHP	CH_3_OH	3	79
6	Fluorescein (10 mol%)	O_2_	CH_3_OH	3	71
7	Fluorescein (10 mol%)	DDQ	CH_3_OH	3	80
8	Fluorescein (10 mol%)	K_2_S_2_O_8_	CH_3_OH	3	75
9	Fluorescein (10 mol%)	TBHP	THF	3	82
10	Fluorescein (10 mol%)	TBHP	DMF	3	58
11	Fluorescein (10 mol%)	TBHP	PhMe	3	74
12	Fluorescein (10 mol%)	TBHP	CH_3_CN	3	80
13	Fluorescein (10 mol%)	TBHP	CH_3_OH	2	82
14	Fluorescein (10 mol%)	TBHP	CH_3_OH	4	89

a Reaction conditions: 2-aminobenzamide 1a (1.0 mmol), benzaldehyde 2a (1.5 mmol), TBHP (2.0 mmol), fluorescein (10 mol%), CH_3_OH (20 mL), blue LED irradiation at room temperature for 3 h.

b Isolated yields.

After determining the optimal reaction conditions, the scope and limitations of each reactant, namely 2-aminobenzamides and aldehydes, were explored. As shown in Table 2, a wide range of aldehydes were reacted with 1a under the optimized reaction conditions. Firstly, we investigated the reaction between 2-aminobenzamide 1a and various aromatic aldehyde under standard condition. When there was no substituent on the benzaldehyde 2a, the yield of the product 3aa could reach 89%. Aromatic aldehydes bearing electron-withdrawing groups (–F, –Cl and –Br) at the *para*-position were converted to the corresponding products (Table 2, 3ab, 3ac and 3af) in 88–92% yields. Instead, aromatic aldehydes substituted by electron-donating groups (–CH_3_ and –OCH_3_) at the *para*-position afforded the target products (Table 2, 3ah and 3ai) in slightly lower yields. When the same substituent was in a different position, the yields of the corresponding products with the substituent at the *para*-position was found to be better than those with the substituent at the *meta*- and *ortho*-position (Table 2, 3ad–3ah). Multi-substituted aromatic aldehyde worked to afford the corresponding product in a lower yield (Table 2, 3aj). This may be due to the presence of two electron-donating groups on the aromatic aldehyde. In addition, heterocyclic aldehydes such as 2-pyridinecarboxaldehyde and 2-thenaldehyde were tested successively and the corresponding products 3ak and 3al were obtained in 81% and 86% yields, respectively. In the case of aliphatic aldehydes such as cyclopropanecarboxaldehyde, isobutyraldehyde and propionaldehyde could also be transformed to the desired products in 47–56% yields (Table 2, 3am–3ao).

**Table 2:** Substrate scope of aldehydes^a^

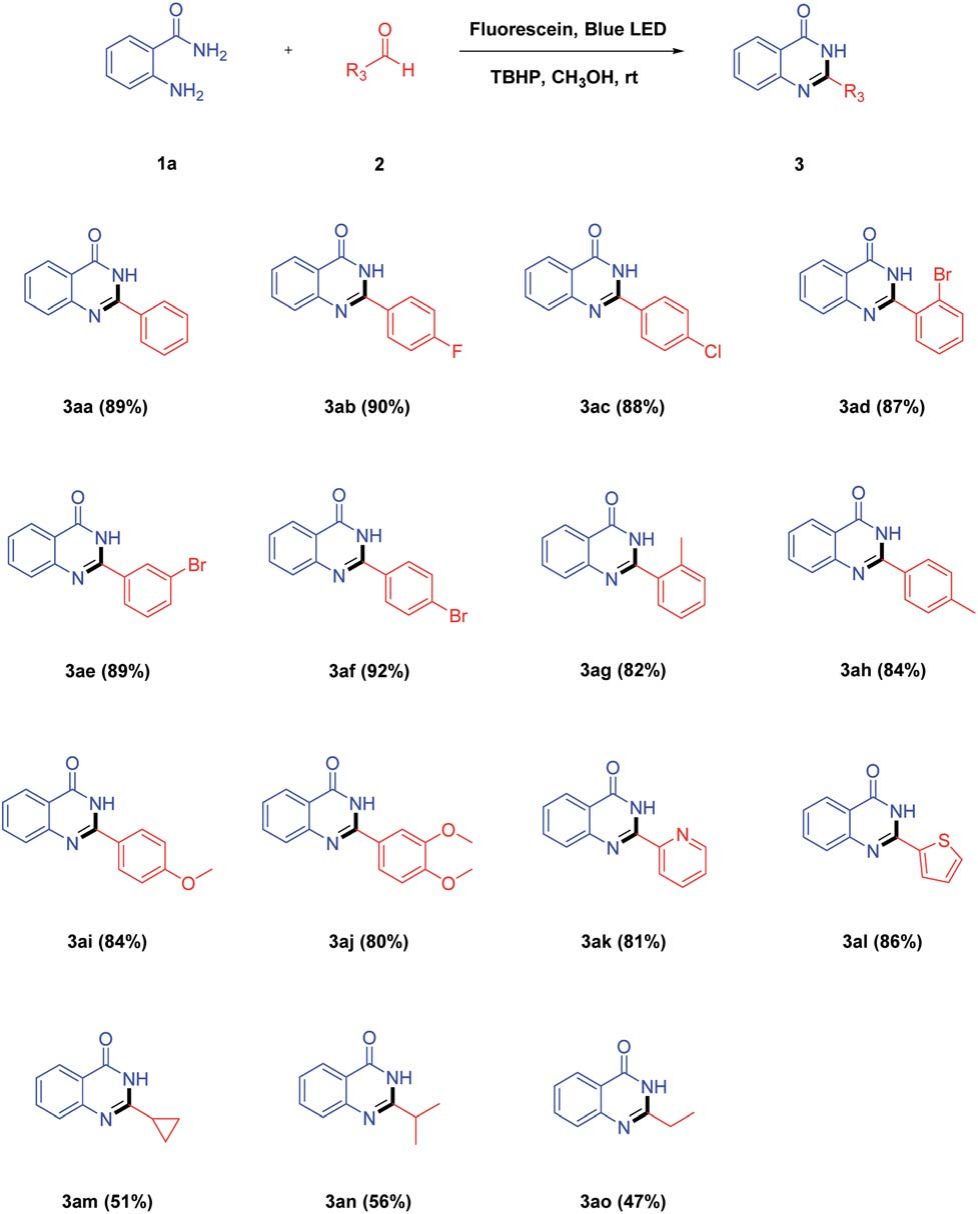

a Reaction conditions: 2-aminobenzamide 1a (1.0 mmol), aldehydes 2 (1.5 mmol), TBHP (2.0 mmol), fluorescein (10 mol%), CH_3_OH (20 mL), blue LED irradiation at room temperature for 3 h.

With the above results in hand, a variety of 2-aminobenzamides were then applied to react with benzaldehyde 2a under the optimized reaction conditions, and the results are summarized in Table 3. It was observed that 2-aminobenzamides bearing electron-withdrawing or electron-donating groups could be smoothly converted to the target products in good yields of 86–92% (Table 3, 3ba–3fa). For the 2-aminobenzamide moiety, the substituent (–Cl) at the 4-position gave the desired product 3ba in 88% yield, and the substituents (–Cl and –Br) at the 5-position gave the corresponding products 3ca and 3da in the yields of 86% and 89%, respectively. The transformations of 2-aminobenzamides that bore electron-donating groups, such as methoxy, gave the corresponding products 3ea in a better yield of 92%. In addition, 2-amino-*N*-methylbenzamide 1f which did not have a free-NH reacted with benzaldehyde 2a affording 3fa in a higher yield at 90%.

**Table 3:** Substrate scope of various 2-aminobenzamides^a^

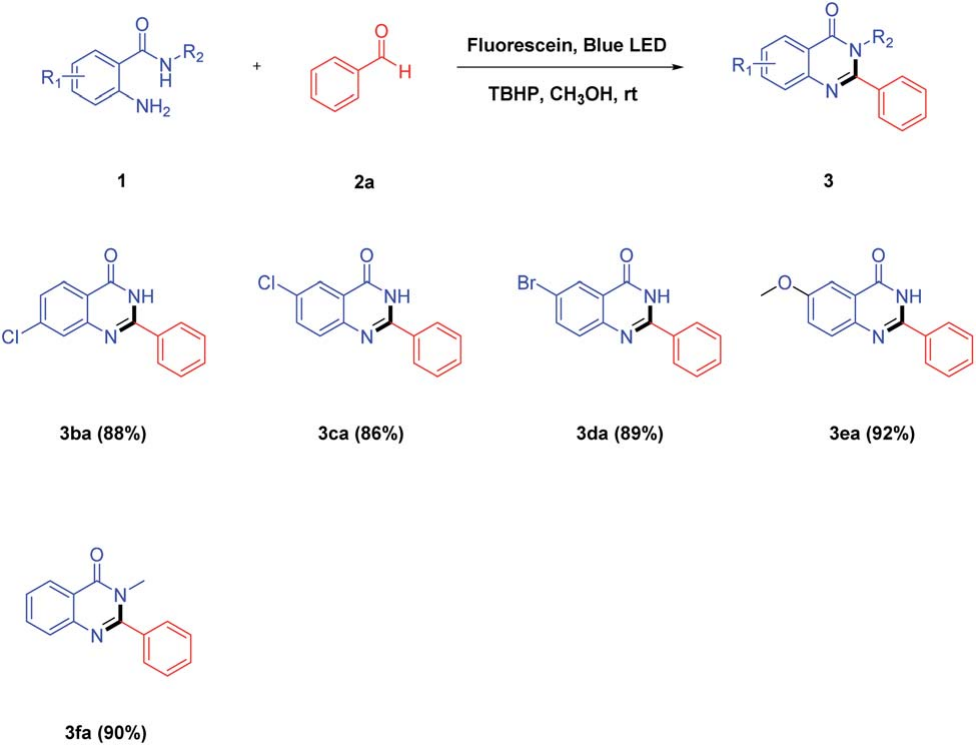

a Reaction conditions: 2-aminobenzamides 1 (1.0 mmol), benzaldehyde 2a (1.5 mmol), TBHP (2.0 mmol), fluorescein (10 mol%), CH_3_OH (20 mL), blue LED irradiation at room temperature for 3 h.

To shed light on the reaction mechanism, some control experiments were conducted (Scheme 2). The first experiment with 2-aminobenzamide 1a and benzaldehyde 2a as the initial material gave 3aa in 89% yield under the optimal reaction conditions (Scheme 2, eqn (1)). As can be seen from eqn (2) and eqn (3), the desired product 3aa was not produced in the system. It is indicated that both blue LED and photocatalyst play an essential role for this reaction. Moreover, we investigated the reaction in the absence of oxidant, and no desired product was detectable in a nitrogen atmosphere (Scheme 2, eqn (4)). When TBHP was added to the above reaction mixture, the desired product 3aa was obtained in a 89% yield. It is indicated that the oxidants have a great influence on the studied reaction, and the reaction affords a good yield in the presence of TBHP (Scheme 2, eqn (5)).

**Scheme 2:**
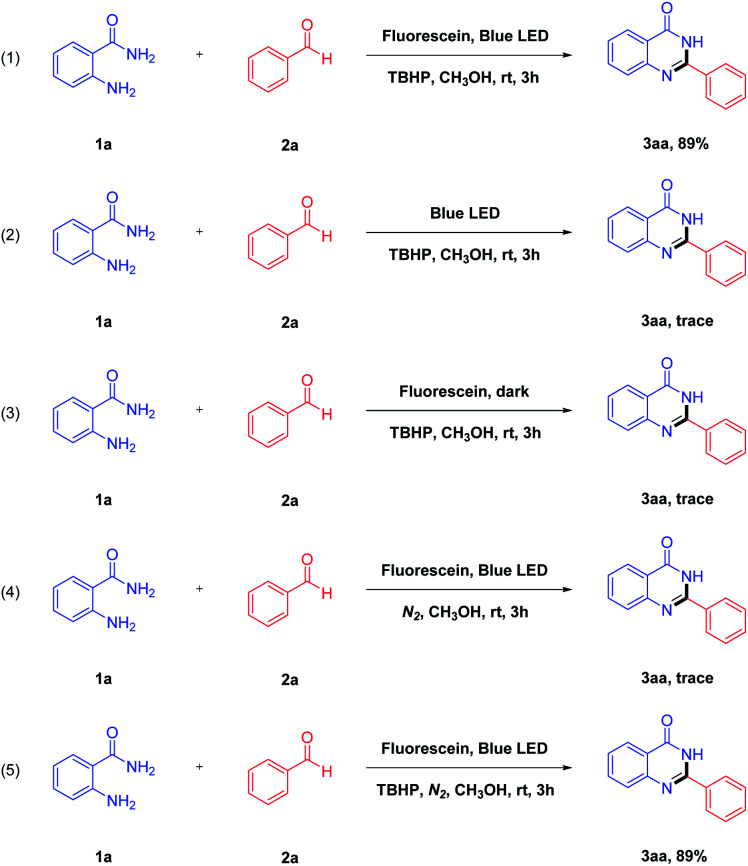
Some control experiments.

Based on the results above and literature reports,^24–26^ a plausible mechanism of the visible light-induced condensation cyclization of 2-aminobenzamide and benzaldehyde is presented in Scheme 3. First, 2-aminobenzamide 1a reacts with benzaldehyde 2a to form the imine intermediates (I), which subsequently generates the intermediates (II) after intramolecular cyclization. Under blue LED irradiation, fluorescein (Fl) generates excited fluorescein* (Fl*) species, and the intermediate (II) is converted to the intermediate (III) *via* single electron transfer. Then, fluorescein radical anion (Fl*^−^) is oxidized to ground state fluorescein (Fl) by TBHP, and the OH^−^ anion and *tert*-butoxy radical (*t*-BuO˙) are released. Next, the reaction of the intermediate (III) with OH^−^ anion affords the intermediate (IV). At last, the desired product 3aa is obtained by deprotonation of intermediate (IV), while *t*-BuOH is also generated from *tert*-butoxy radical (*t*-BuO˙).

**Scheme 3:**
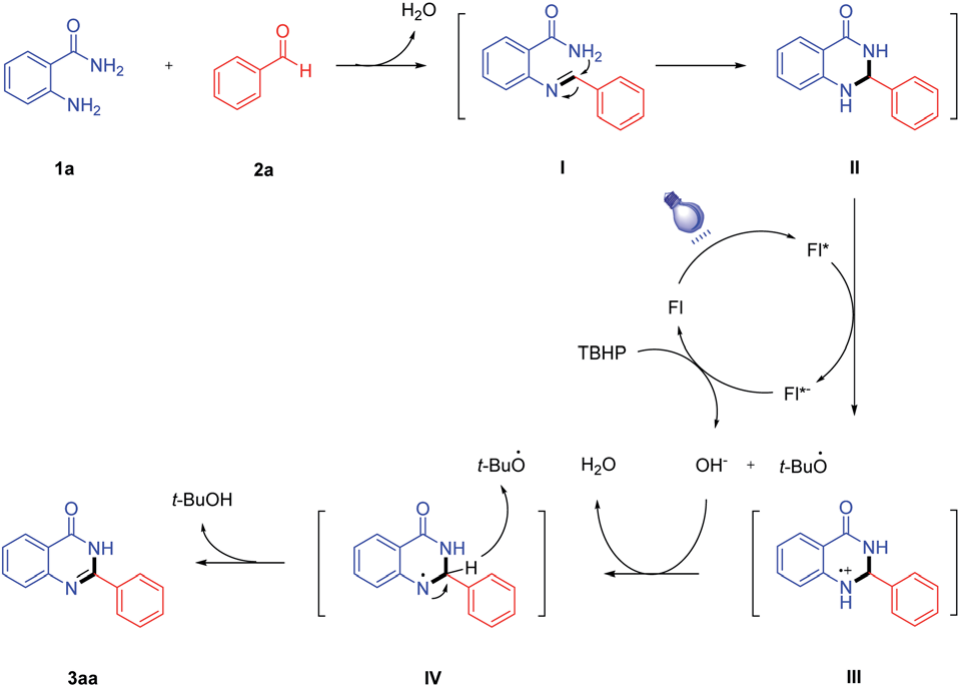
A plausible reaction mechanism.

## Conclusions

In summary, we successfully developed a novel and straightforward approach for synthesis of quinazolin-4(3*H*)-ones *via* visible light-induced condensation cyclization of 2-aminobenzamides and aldehydes using fluorescein as photocatalyst and TBHP as the oxidant. The reactions proceeded smoothly, affording the corresponding quinazolin-4(3*H*)-ones in high to excellent yields with good functional group tolerance. In addition, a plausible reaction mechanism was proposed based on the control experiments and literature reports. The application of this method for preparation of other useful heterocycles is underway in our laboratory.

## Conflicts of interest

There are no conflicts to declare.

## Supplementary Material

RA-012-D1RA07944A-s001
