# Supplementary material for: Straightforward synthesis of quinazolin-4(3H)-ones via visible light-induced condensation cyclization
Source: RSC Adv. 2022 Jan 7;12(3):1494–8. doi: 10.1039/d1ra07944a (PMC8978923; doi:10.1039/d1ra07944a)
Supplement: RA-012-D1RA07944A-s001 [file RA-012-D1RA07944A-s001.pdf]

Electronic Supplementary Material (ESI) for RSC Advances.
This journal is © The Royal Society of Chemistry 2021

Electronic Supplementary Information

Straightforward synthesis of quinazolin-4(3*H*)-ones via visible light-induced condensation cyclization

Wuji Sun,[a] Xue Ma,[a] Yuqi Pang,[a] Letian Ying,[a] Lifeng Zhao,[a] Qidi Zhong,[a] Chunyan Liu*[a] and Qiangwen Fan*[b]

[a] School of Pharmacy, North China University of Science and Technology, Tangshan, 063210, China

[b] School of Chemistry, Biology and Material Science, East China University of Technology, Nanchang, 330013, China

*Corresponding author: chunyanliu@ncst.edu.cn; fanqw2019@ecut.edu.cn

Tables of contents

1. General Information S2

2. General procedure for synthesis of products **3** S2

3. NMR spectra and analysis of products **3aa-3ao** S3

4. NMR spectra and analysis of products **3ba-3fa** S15

General Information

All starting materials and the reagents were purchased from commercial sources, and the reagents were used without further purification. The reactions were monitored by thin layer chromatography (TLC) on 0.25 mm silica gel plates (GF254), and the products were purified by column chromatography on silica gel (200-300 mesh). ^{1}H NMR and ^{13}C NMR spectra were recorded on a Bruker Avance 400 spectrometer operating at 400 MHz and 100 MHz in DMSO-d_6. The following abbreviations were used to describe peak splitting patterns when appropriate: s = singlet, d = doublet, t = triplet, m = multiple. Coupling constants (J) were reported in Hertz (Hz). Mass spectra (ESI-HRMS) were recorded using an Agilent Accurate-Mass Q-TOF LC/MS 6520 instrument with an ESI source. CREE XPE 30 W blue LED were used as light sources.

General procedure for synthesis of products 3

To a flat quartz glass jar was charged 2-aminobenzamides **1** (1.0 mmol), aldehydes **2** (1.5 mmol), TBHP (2.0 mmol), fluorescein (10 mol%) and methanol (20 mL). The reaction mixture was irradiated with CREE XPE 30 W blue LED (approximately 10 cm away from the light source) under two mini fans at room temperature for 3 h. Upon the reaction completion, the mixture was evaporated under reduced pressure to give the crude product, and purification of the crude product by silica gel column chromatography afforded the corresponding products **3**.

NMR spectra and analysis of products 3aa-3ao

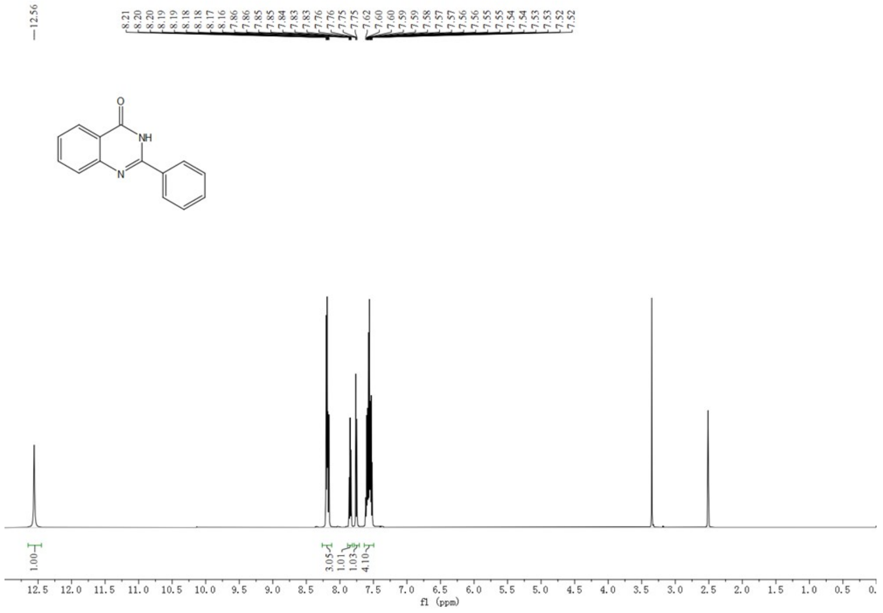

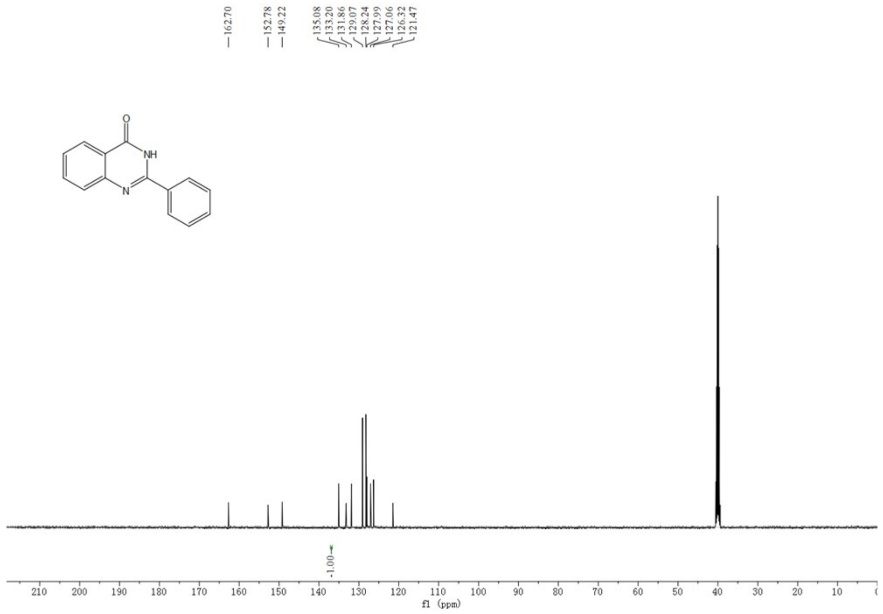

2-Phenylquinazolin-4(3*H*)-one (3aa).[1] 89% yield as a white solid; mp 229-231℃ (lit.[1] mp 230-232℃); ^{1}H NMR (400 MHz, DMSO-d_6): δ (ppm) 12.56 (s, 1H), 8.21-8.16 (m, 3H), 7.86-7.83 (m, 1H), 7.76 (dd, J = 8.2, 1.2 Hz, 1H), 7.62-7.52 (m, 4H); ^{13}C NMR (100 MHz, DMSO-d_6): δ (ppm) 162.70, 152.78, 149.22, 135.08, 133.20, 131.86, 129.07, 128.24, 127.99, 127.06, 126.32, 121.47; HRMS (ESI) m/z: calcd for $C_{14}H_{11}N_2O$ [M + H]$^+$ 223.0866, found 223.0867.

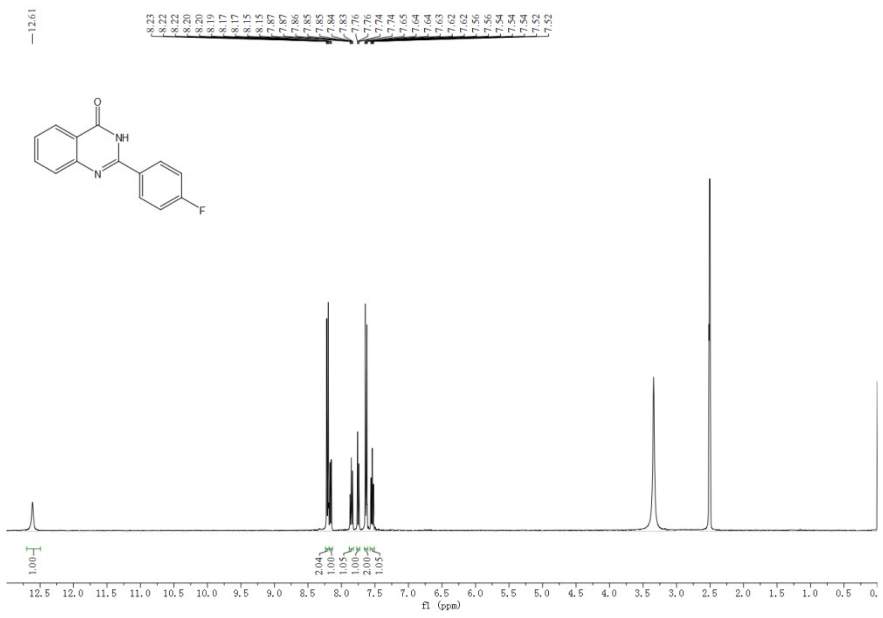

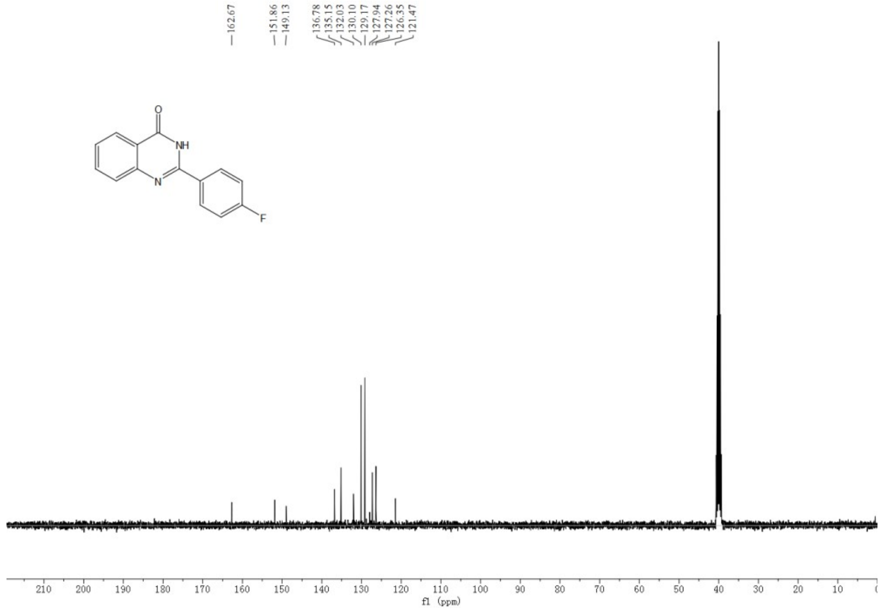

2-(4-Fluorophenyl)quinazolin-4(3*H*)-one (3ab).[1] 90% yield as a yellow solid; mp 285-287°C (lit.[1] mp 284-286°C); ^{1}H NMR (400 MHz, DMSO-d_6): δ (ppm) 12.61 (s, 1H), 8.23-8.19 (m, 2H), 8.16 (dd, J = 7.9, 1.5 Hz, 1H), 7.87-7.83 (m, 1H), 7.75 (dd, J = 8.3, 1.2 Hz, 1H), 7.65-7.62 (m, 2H), 7.56-7.52 (m, 1H); ^{13}C NMR (100 MHz, DMSO-d_6): δ (ppm) 162.67, 151.86, 149.13, 136.78, 135.15, 132.03, 130.10, 129.17, 127.94, 127.26, 126.35, 121.47; HRMS (ESI) m/z: calcd for $C_{14}H_{10}FN_2O$ $[M + H]^+$ 241.0772, found 241.0768.

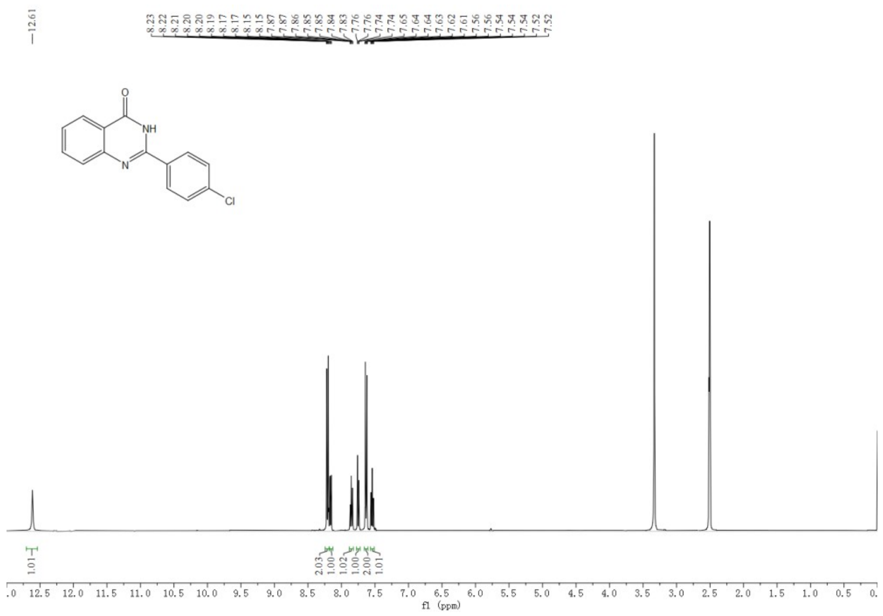

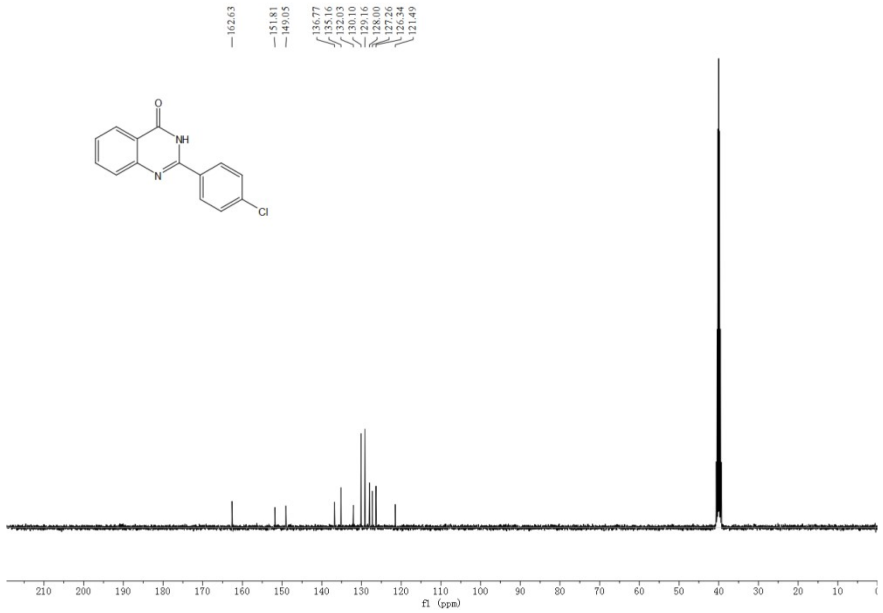

2-(4-Chlorophenyl)quinazolin-4(3*H*)-one (3ac).[1] 86% yield as a white solid; mp ＞300℃ (lit.[1] mp ＞300℃); ^{1}H NMR (400 MHz, DMSO-d_6): δ (ppm) 12.61 (s, 1H), 8.23-8.19 (m, 2H), 8.16 (dd, J = 8.0, 1.5 Hz, 1H), 7.87-7.83 (m, 1H), 7.75 (dd, J = 8.3, 1.2 Hz, 1H), 7.65-7.61 (m, 2H), 7.56-7.52 (m, 1H); ^{13}C NMR (100 MHz, DMSO-d_6): δ (ppm) 162.63, 151.81, 149.05, 136.77, 135.16, 132.03, 130.10, 129.16, 128.00, 127.26, 126.34, 121.49; HRMS (ESI) m/z: calcd for $C_{14}H_{10}ClN_2O$ [M + H]$^+$ 257.0476, found 257.0479.

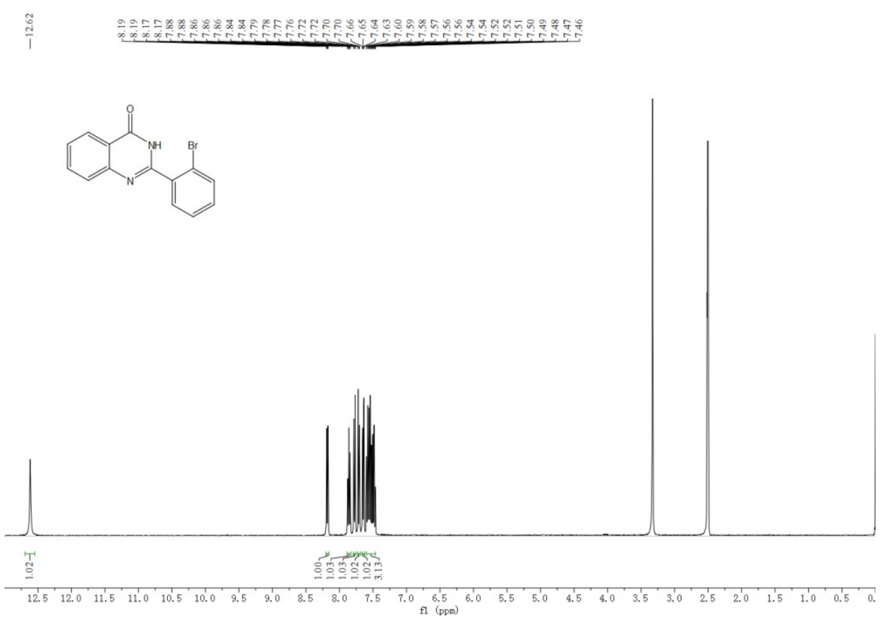

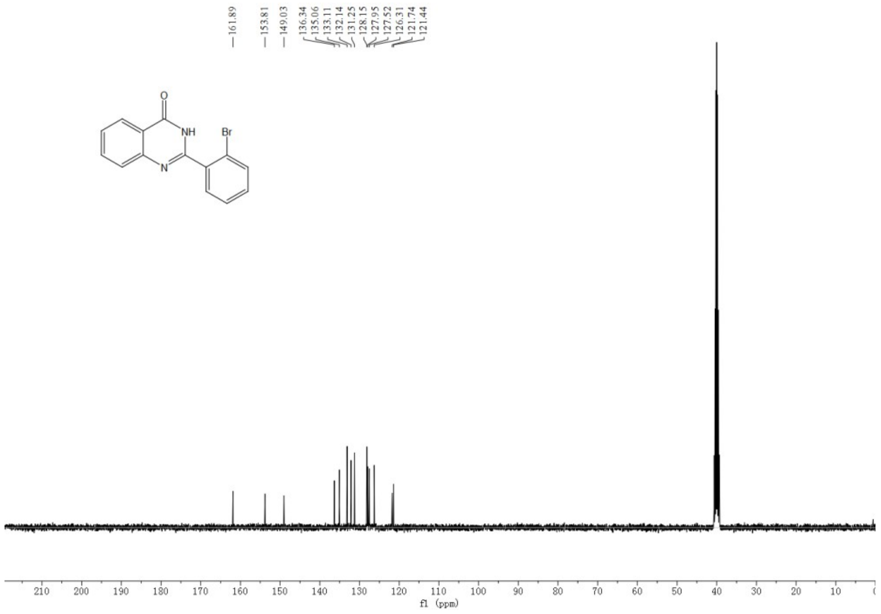

2-(2-Bromophenyl)quinazolin-4(3*H*)-one (3ad).[2] 86% yield as a white solid; mp 174-176°C (lit.[2] mp 175-177°C); ^{1}H NMR (400 MHz, DMSO-d_6): δ (ppm) 12.62 (s, 1H), 8.18 (dd, J = 7.9, 1.6 Hz, 1H), 7.88-7.84 (m, 1H), 7.78 (dd, J = 7.9, 1.3 Hz, 1H), 7.71 (dd, J = 8.3, 1.1 Hz, 1H), 7.65 (dd, J = 7.5, 1.9 Hz, 1H), 7.60-7.46 (m, 3H); ^{13}C NMR (100 MHz, DMSO-d_6): δ (ppm) 161.89, 153.81, 149.03, 136.34, 135.06, 133.11, 132.14, 131.25, 128.15, 127.95, 127.52, 126.31, 121.74, 121.44; HRMS (ESI) m/z: calcd for $C_{14}H_{10}BrN_2O$ $[M + H]^+$ 300.9971, found 300.9972.

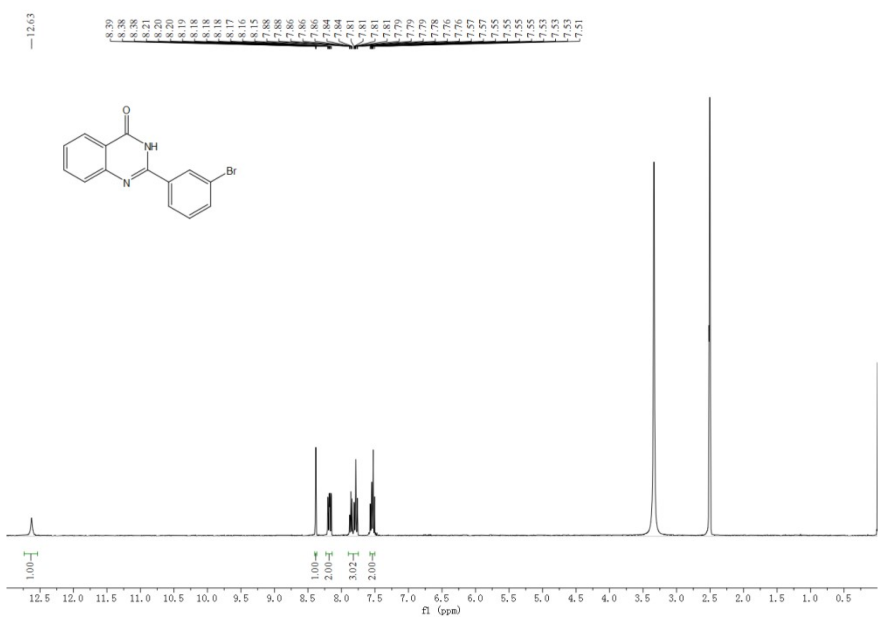

2-(3-Bromophenyl)quinazolin-4(3*H*)-one (3ae).[3] 89% yield as a yellow solid; mp 295-296°C (lit.[3] mp 295-296°C); ^{1}H NMR (400 MHz, DMSO-d_6): δ (ppm) 12.63 (s, 1H), 8.38 (t, J = 1.9 Hz, 1H), 8.21-8.15 (m, 2H), 7.88-7.76 (m, 3H), 7.57-7.51 (m, 2H); HRMS (ESI) m/z: calcd for $C_{14}H_{10}BrN_2O$ [M + H]$^+$ 300.9971, found 300.9974.

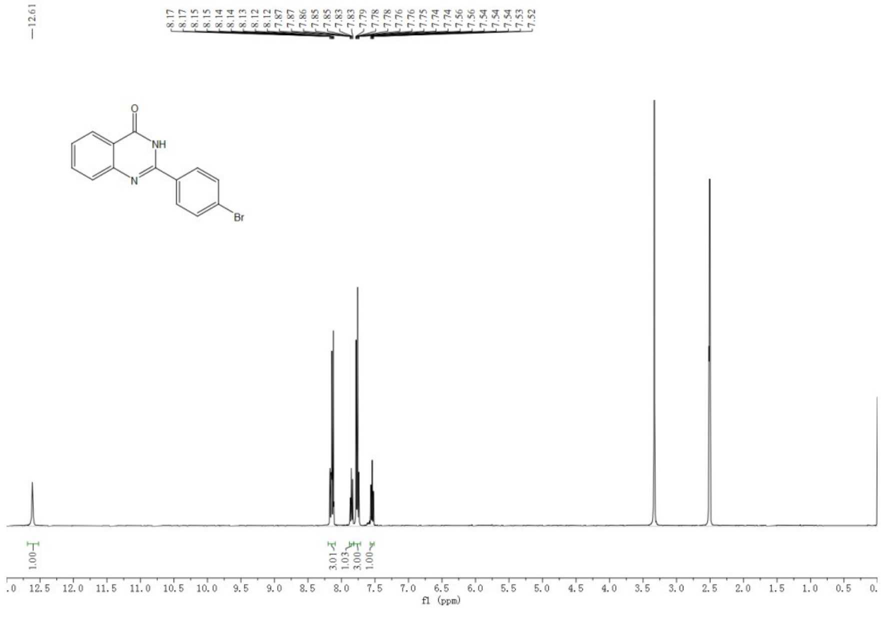

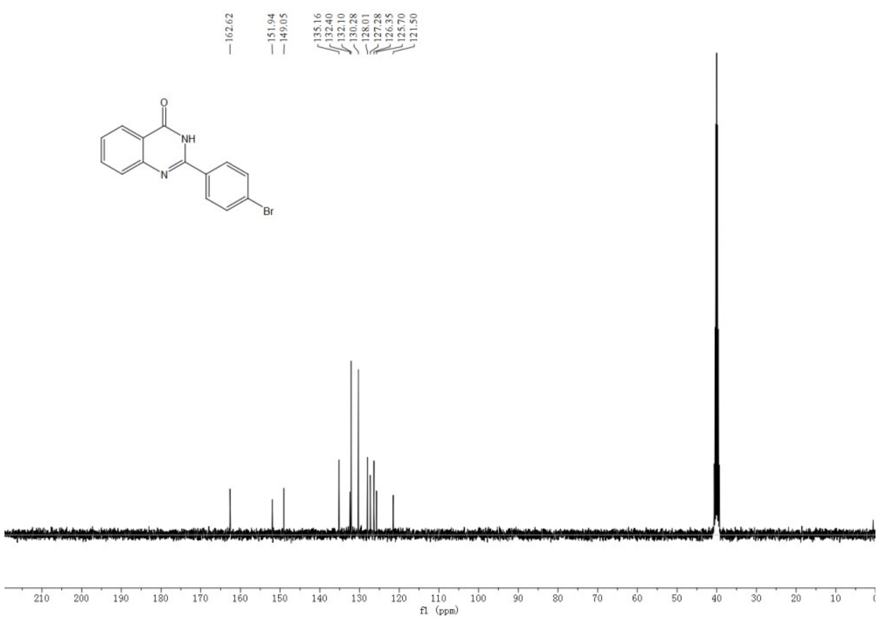

2-(4-Bromophenyl)quinazolin-4(3*H*)-one (3af).[1] 92% yield as a yellow solid; mp 292-293°C (lit.[1] mp 292-294°C); ^{1}H NMR (400 MHz, DMSO-d_6): δ (ppm) 12.61 (s, 1H), 8.17-8.12 (m, 3H), 7.87-7.83 (m, 1H), 7.79-7.74 (m, 3H), 7.56-7.52 (m, 1H); ^{13}C NMR (100 MHz, DMSO-d_6): δ (ppm) 162.62, 151.94, 149.05, 135.16, 132.40, 132.10, 130.28, 128.01, 127.28, 126.35, 125.70, 121.50; HRMS (ESI) m/z: calcd for $C_{14}H_{10}BrN_2O$ $[M + H]^+$ 300.9971, found 300.9969.

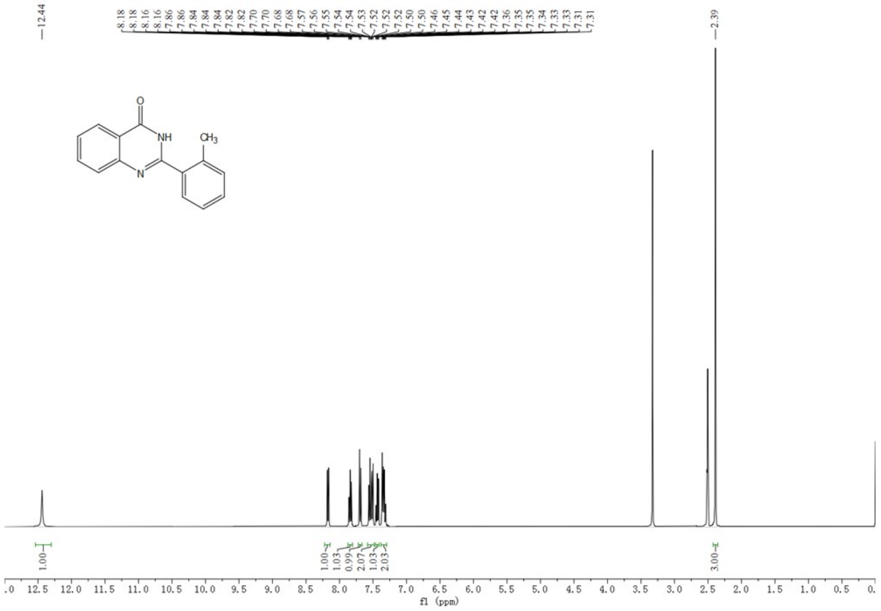

2-(2-Methylphenyl)quinazolin-4(3*H*)-one (3ag).[4] 83% yield as a white solid; mp 214-215°C (lit.[4] mp 216-218°C); ^{1}H NMR (400 MHz, DMSO-d_6): δ (ppm) 12.44 (s, 1H), 8.17 (dd, J = 7.9,

1.6 Hz, 1H), 7.86-7.82 (m, 1H), 7.69 (dd, J = 8.3, 1.2 Hz, 1H), 7.57-7.50 (m, 2H), 7.44 (td, J = 7.4, 1.5 Hz, 1H), 7.36-7.31 (m, 2H), 2.39 (s, 3H); HRMS (ESI) m/z: calcd for $C_{15}H_{13}N_2O$ [M + H]$^+$ 237.1022, found 237.1022.

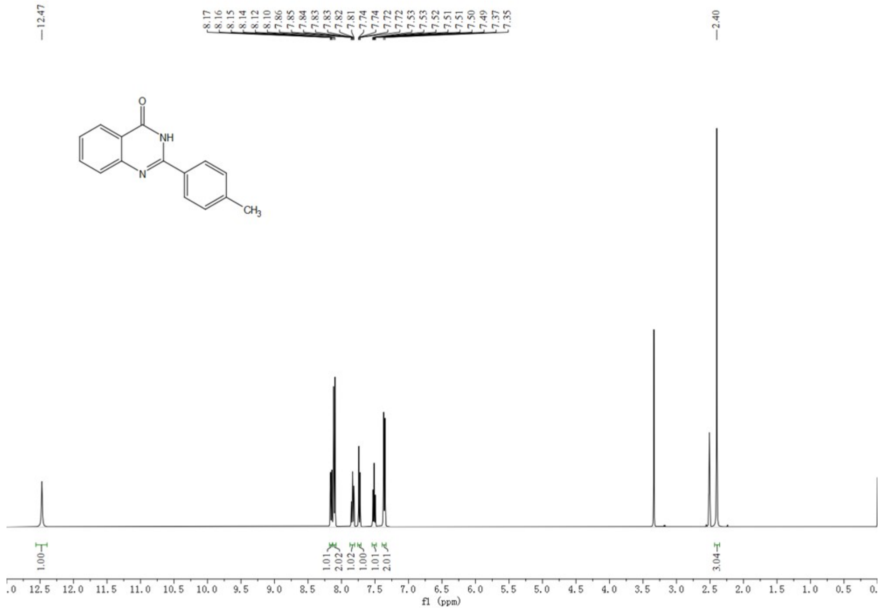

2-(4-Methylphenyl)quinazolin-4(3*H*)-one (3ah).[4] 81% yield as a white solid; mp 240-242°C (lit.[4] mp 241-243°C); ^{1}H NMR (400 MHz, DMSO-d_6): δ (ppm) 12.47 (s, 1H), 8.15 (dd, J = 7.9, 1.5 Hz, 1H), 8.11 (d, J = 8.3 Hz, 2H), 7.86-7.81 (m, 1H), 7.73 (dd, J = 8.3, 1.2 Hz, 1H), 7.53-7.49 (m, 1H), 7.36 (d, J = 8.3 Hz, 2H), 2.40 (s, 3H); HRMS (ESI) m/z: calcd for $C_{15}H_{13}N_2O$ [M + H]$^+$ 237.1022, found 237.1020.

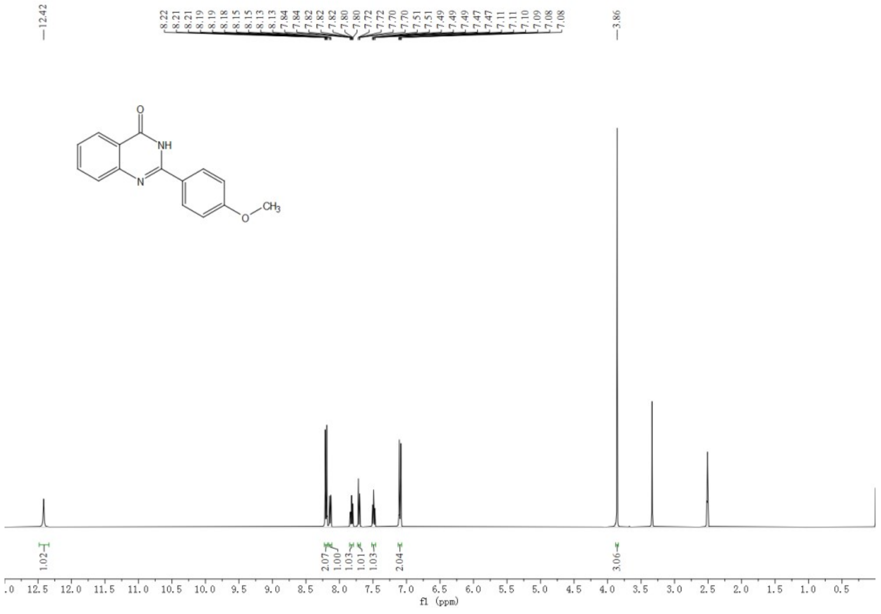

2-(4-Methoxyphenyl)quinazolin-4(3*H*)-one (3ai).[2] 82% yield as a white solid; mp 228-230°C (lit.[2] mp 230-232°C); ^{1}H NMR (400 MHz, DMSO-d_6): δ (ppm) 12.42 (s, 1H), 8.22-8.18 (m, 2H), 8.14 (dd, J = 8.0, 1.5 Hz, 1H), 7.84-7.80 (m, 1H), 7.71 (dd, J = 8.4, 1.2 Hz, 1H), 7.51-7.47 (m, 1H), 7.11-7.08 (m, 2H), 3.86 (s, 3H); HRMS (ESI) m/z: calcd for $C_{15}H_{13}N_2O_2$ $[M + H]^+$ 253.0972, found 253.0976.

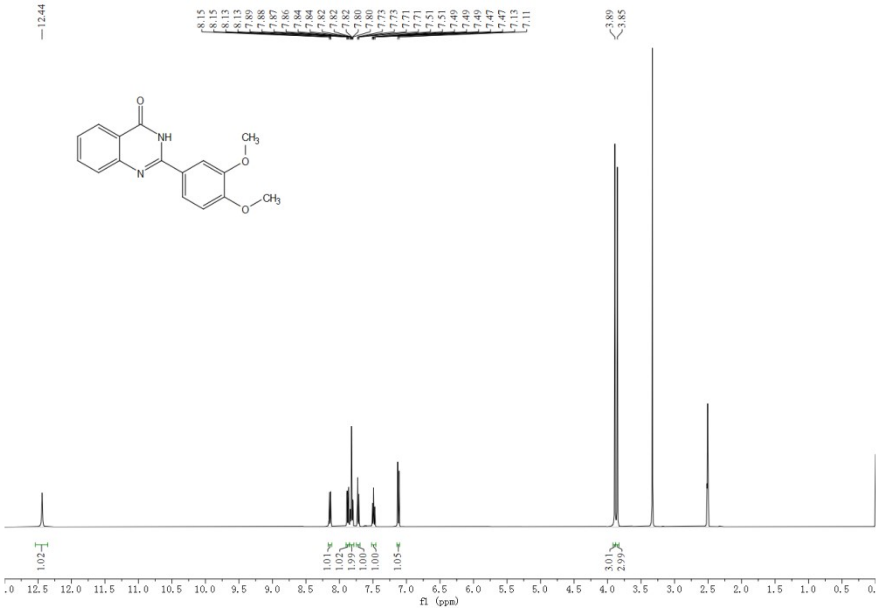

2-**(3,4-Dimethoxyphenyl)quinazolin-4(3*H*)-one (3aj)**.[5] 85% yield as a white solid; mp 241-242°C (lit.[5] mp 242-243°C); ^{1}H NMR (400 MHz, DMSO-d_6): δ (ppm) 12.44 (s, 1H), 8.14 (dd, J =

8.0, 1.5 Hz, 1H), 7.88 (dd, J = 8.5, 2.2 Hz, 1H), 7.84-7.80 (m, 2H), 7.72 (dd, J = 8.3, 1.2 Hz, 1H), 7.51-7.47 (m, 1H), 7.12 (d, J = 8.5 Hz, 1H), 3.89 (s, 3H), 3.85 (s, 3H); HRMS (ESI) m/z: calcd for $C_{16}H_{15}N_2O_3$ $[M + H]^+$ 253.0972, found 253.0976.

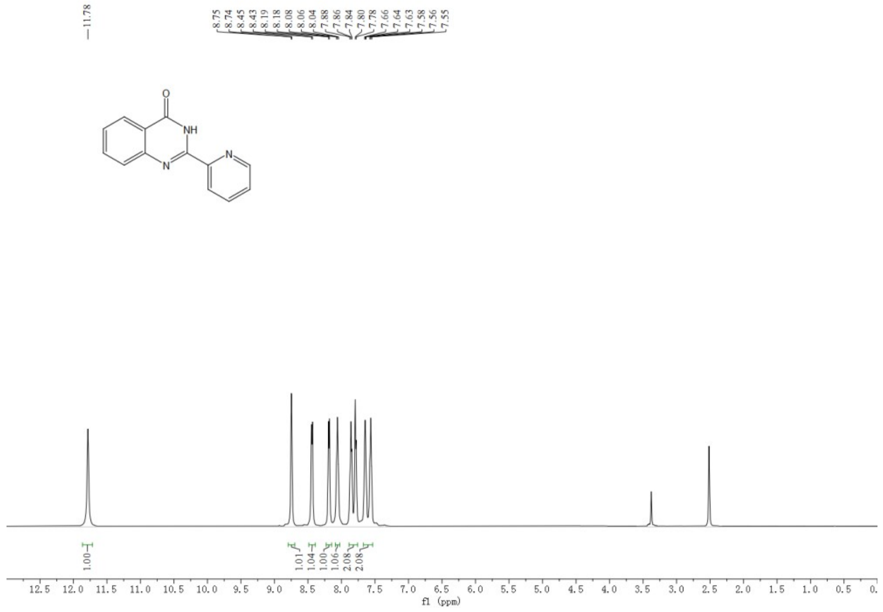

2-(Pyridin-2-yl)quinazolin-4(3*H*)-one (3ak).[4] 81% yield as a white solid; mp 171-173°C (lit.[4] mp 170-173°C); ^{1}H NMR (400 MHz, DMSO-d_6): δ (ppm) 11.78 (s, 1H), 8.75 (d, J = 4.8 Hz, 1H), 8.44 (d, J = 7.9 Hz, 1H), 8.19 (d, J = 7.9 Hz, 1H), 8.06 (t, J = 7.9 Hz, 1H), 7.88-7.78 (m, 2H), 7.66-7.55 (m, 2H); HRMS (ESI) m/z: calcd for $C_{13}H_{10}N_3O$ $[M + H]^+$ 224.0818, found 224.0821.

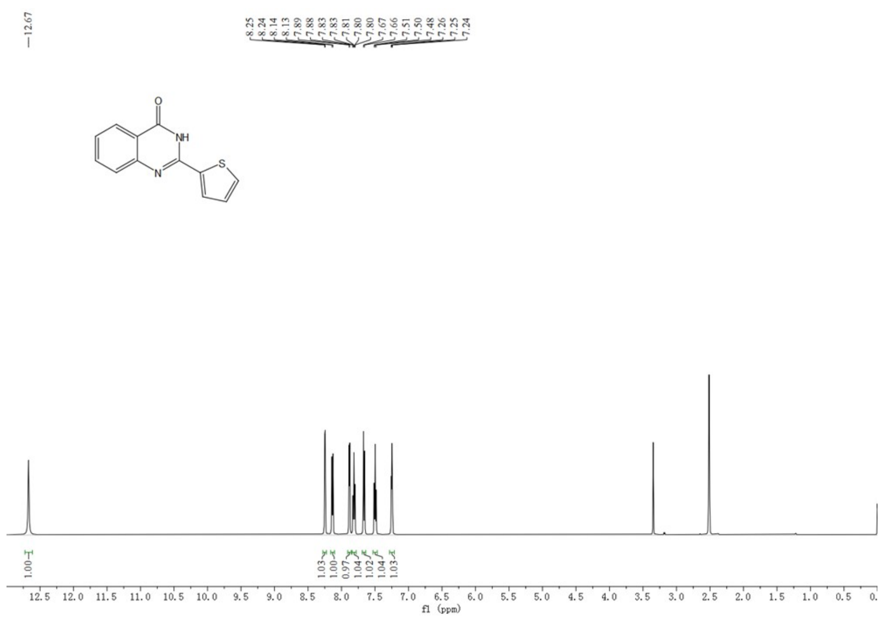

2-(Thiophene-2-yl)quinazolin-4(3*H*)-one (3al).[1] 86% yield as a white solid; mp 220-222℃ (lit.[1] mp 221-222℃); ^{1}H NMR (400 MHz, DMSO-d_6): δ (ppm) 12.67 (s, 1H), 8.25 (d, J = 4.4 Hz, 1H), 8.14 (d, J = 7.8 Hz, 1H), 7.88 (d, J = 5.0 Hz, 1H), 7.83-7.80 (m, 1H), 7.66 (d, J = 8.1 Hz, 1H), 7.50 (t, J = 7.8 Hz, 1H), 7.25 (t, J = 4.4 Hz, 1H); HRMS (ESI) m/z: calcd for $C_{12}H_9N_2OS$ [M + H]$^+$ 229.0430, found 229.0432.

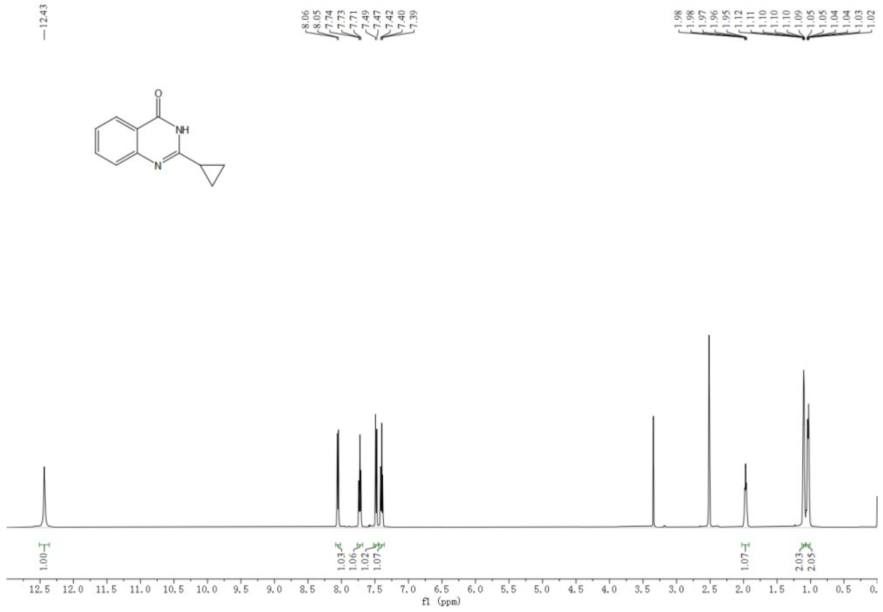

2-Cyclopropylquinazolin-4(3*H*)-one (3am).[6] 51% yield as a white solid; mp 231-232℃ (lit.[6] mp 233-234℃); ^{1}H NMR (400 MHz, DMSO-d_6): δ (ppm) 12.43 (s, 1H), 8.05 (d, J = 7.8 Hz, 1H), 7.73 (t, J = 7.7 Hz, 1H), 7.48 (d, J = 8.1 Hz, 1H), 7.40 (t, J = 7.5 Hz, 1H), 1.97 (m, 1H), 1.10 (m, 2H), 1.04 (m, 2H); HRMS (ESI) m/z: calcd for $C_{11}H_{11}N_2O$ [M + H]$^+$ 187.0866, found 187.0869.

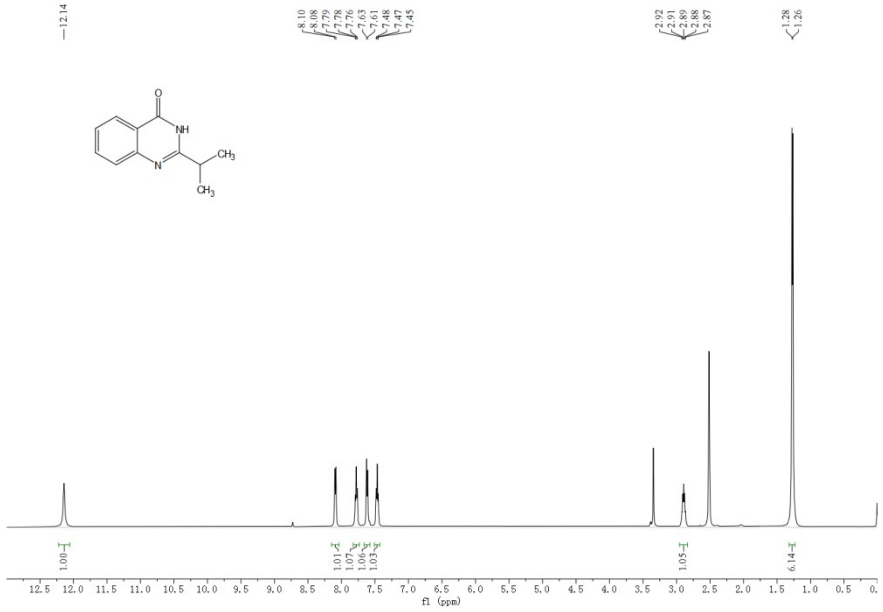

2-Isopropylquinazolin-4(3*H*)-one (3an).[7] 56% yield as a white solid; mp 222-224°C (lit.[7] mp 225-228°C); ^{1}H NMR (400 MHz, DMSO-d_6): δ (ppm) 12.14 (s, 1H), 8.09 (d, *J* = 7.8 Hz, 1H), 7.78 (t, *J* = 7.7 Hz, 1H), 7.62 (d, *J* = 8.2 Hz, 1H), 7.47 (t, *J* = 7.5 Hz, 1H), 2.89 (m, 1H), 1.27 (d, *J* = 6.9 Hz, 6H); HRMS (ESI) m/z: calcd for $C_{11}H_{13}N_2O$ $[M + H]^+$ 189.1022, found 189.1026.

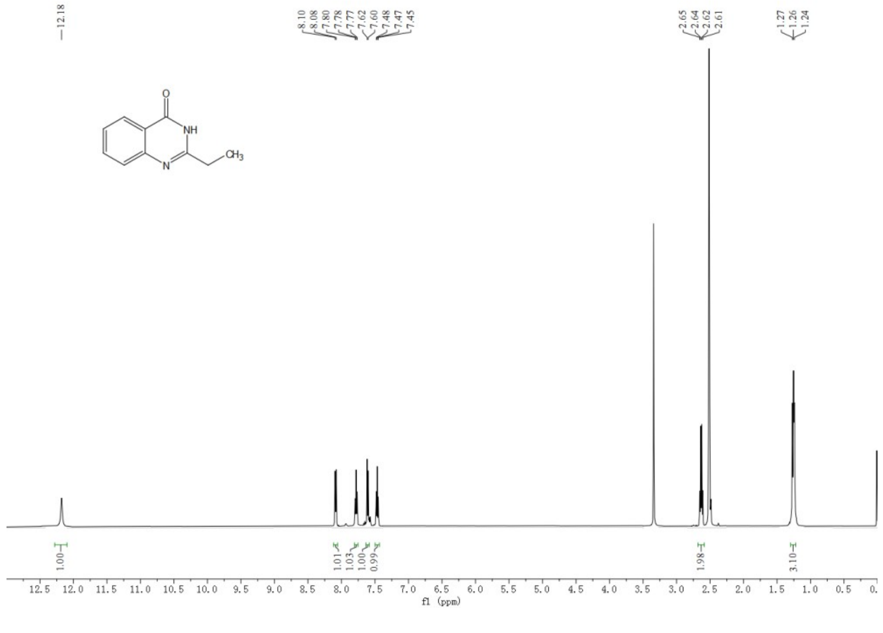

2-Ethylquinazolin-4(3*H*)-one (3ao).[1] 47% yield as a white solid; mp 226-227°C (lit.[1] mp 228-230°C); ^{1}H NMR (400 MHz, DMSO-d_6): δ (ppm) 12.18 (s, 1H), 8.09 (d, *J* = 7.9 Hz, 1H), 7.78 (t, *J* = 7.7 Hz, 1H), 7.61 (d, *J* = 8.1 Hz, 1H), 7.47 (t, *J* = 7.6 Hz, 1H), 2.63 (q, *J* = 7.5 Hz, 2H), 1.26 (t, *J* = 7.5

Hz, 3H); HRMS (ESI) m/z: calcd for $C_{10}H_{11}N_2O$ $[M + H]^+$ 175.0866, found 175.0868.

NMR spectra and analysis of products 3ba-3fa

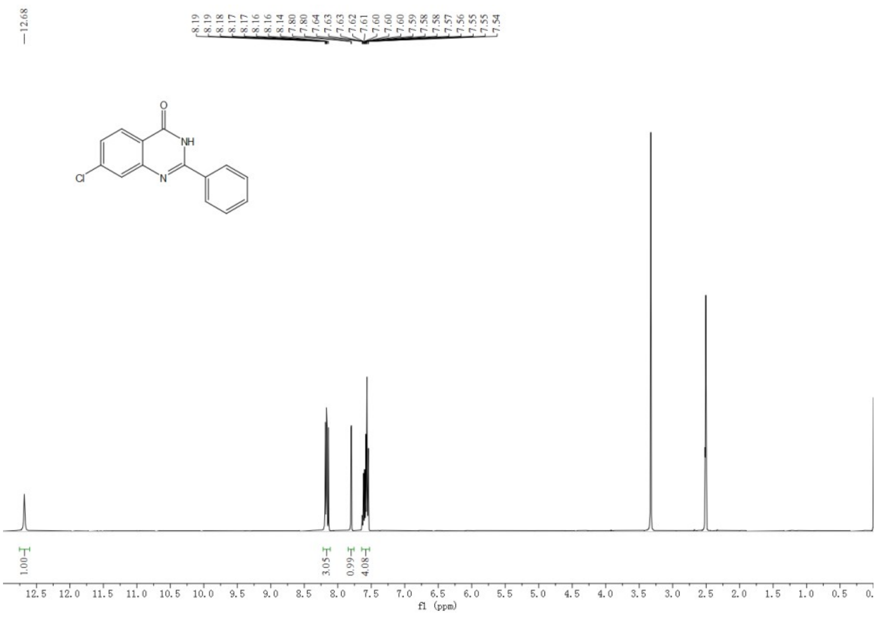

7-Chloro-2-phenylquinazolin-4(3*H*)-one (3ba).[3] 88% yield as a white solid; mp 283-285℃ (lit.[3] mp 287-288℃); ^{1}H NMR (400 MHz, DMSO-d_6): δ (ppm) 12.68 (s, 1H), 8.19-8.14 (m, 3H), 7.80 (d, J = 2.0 Hz, 1H), 7.64-7.54 (m, 4H); HRMS (ESI) m/z: calcd for $C_{14}H_{10}ClN_2O$ $[M + H]^+$ 257.0476, found 257.0475.

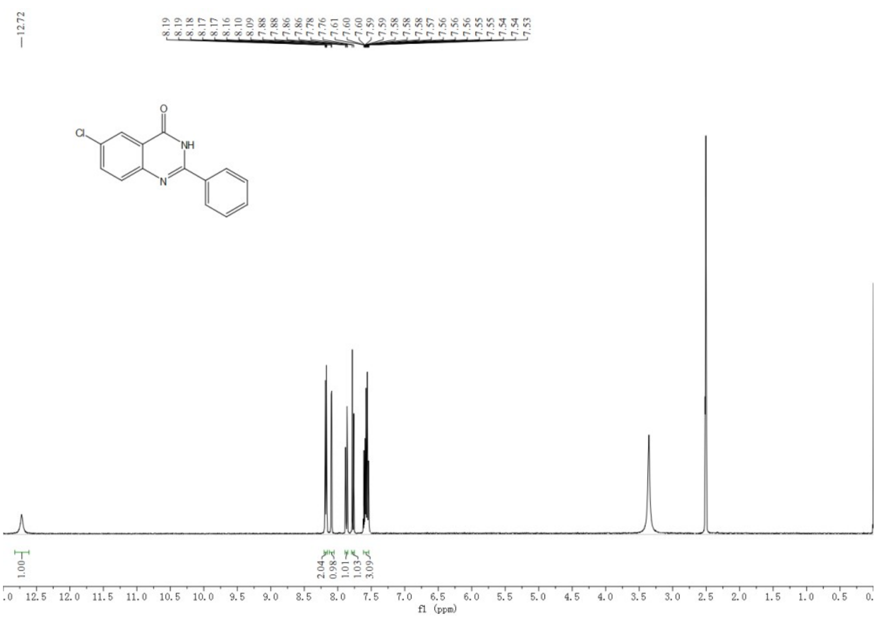

6-Chloro-2-phenylquinazolin-4(3*H*)-one (3ca).[3] 86% yield as a white solid; mp 295-296℃ (lit.[3] mp 296-297℃); ^{1}H NMR (400 MHz, DMSO-d_6): δ (ppm) 12.72 (s, 1H), 8.19-8.16 (m, 2H), 8.09

(d, J = 2.5 Hz, 1H), 7.87 (dd, J = 8.7, 2.5 Hz, 1H), 7.77 (d, J = 8.7 Hz, 1H), 7.61-7.53 (m, 3H); HRMS (ESI) m/z: calcd for $C_{14}H_{10}ClN_2O$ $[M + H]^+$ 257.0476, found 257.0478.

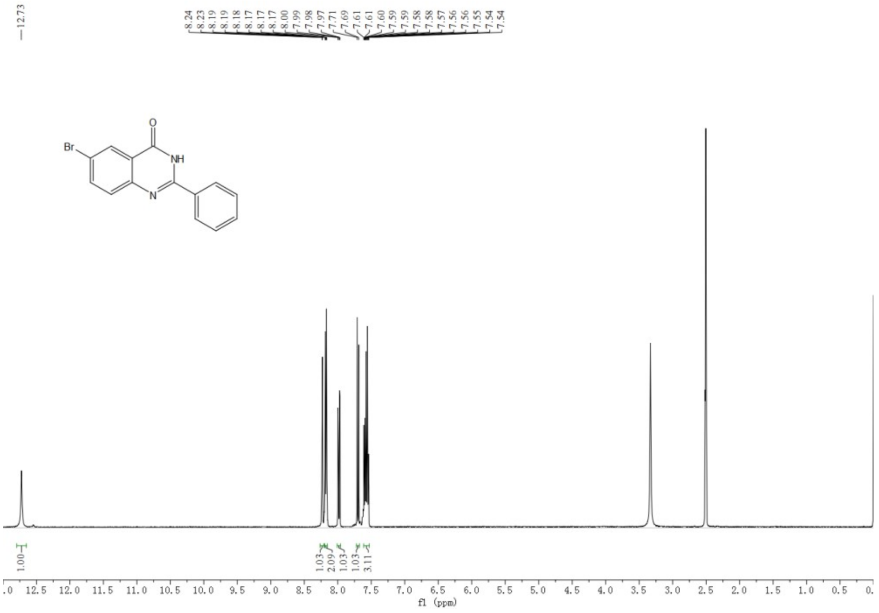

6-Bromo-2-phenylquinazolin-4(3*H*)-one (3da).[4] 89% yield as a yellow solid; mp 285-287°C (lit.[4] mp 284-286°C); ^{1}H NMR (400 MHz, DMSO-d_6): δ (ppm) 12.73 (m, 1H), 8.23 (d, J = 2.3 Hz, 1H), 8.19-8.17 (m, 2H), 7.98 (dd, J = 8.7, 2.3 Hz, 1H), 7.70 (d, J = 8.7 Hz, 1H), 7.61-7.54 (m, 3H); HRMS (ESI) m/z: calcd for $C_{14}H_{10}BrN_2O$ $[M + H]^+$ 300.9971, found 300.9973.

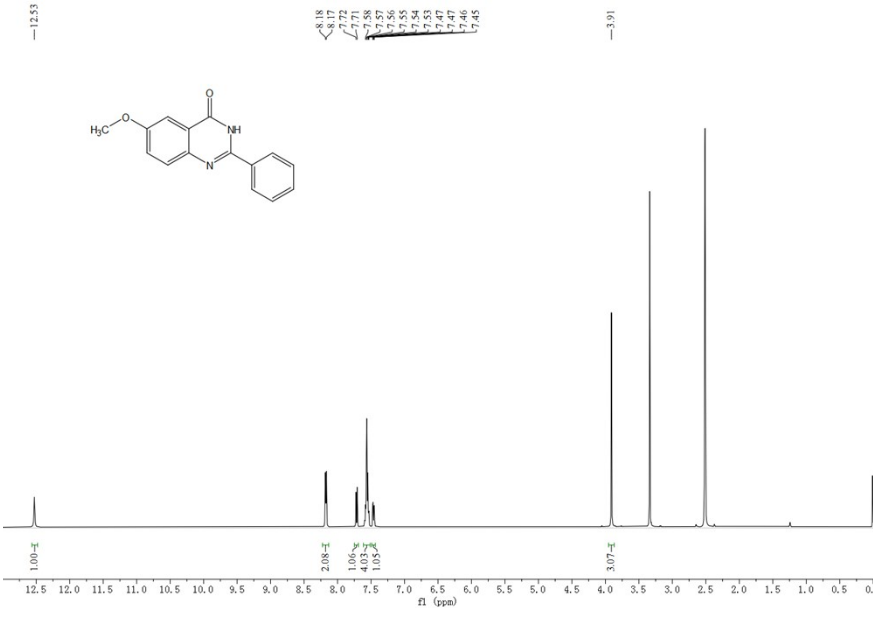

6-Methoxy-2-phenylquinazolin-4(3*H*)-one (3ea).[1] 92% yield as a white solid; mp 249-251°C

(lit.[1] mp 246-247°C); ^{1}H NMR (400 MHz, DMSO-d_6): δ (ppm) 12.53 (s, 1H), 8.17 (d, J = 7.3 Hz, 2H), 7.72 (d, J = 8.9 Hz, 1H), 7.58-7.53 (m, 4H), 7.46 (dd, J = 8.9, 2.9 Hz, 1H), 3.91 (s, 3H); HRMS (ESI) m/z: calcd for $C_{15}H_{13}N_2O_2$ [M + H]$^+$ 253.0972, found 253.0975.

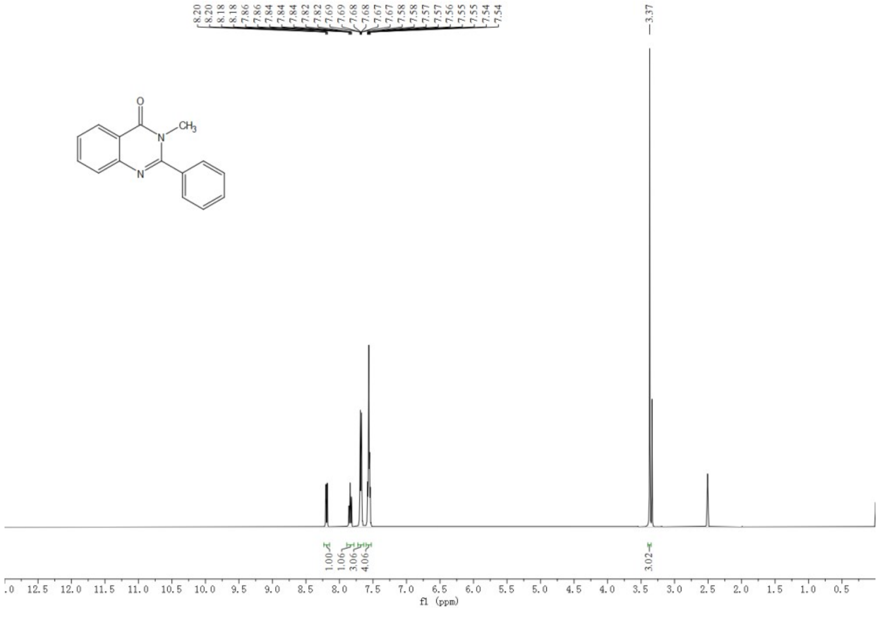

3-Methyl-2-phenylquinazolin-4(3*H*)-one (3fa).[4] 90% yield as a white solid; mp 123-125°C (lit.[4] mp 125-127°C); ^{1}H NMR (400 MHz, DMSO-d_6): δ (ppm) 8.19 (dd, J = 8.0, 1.5 Hz, 1H), 7.86-7.82 (m, 1H), 7.69-7.67 (m, 3H), 7.58-7.54 (m, 4H), 3.37 (s, 3H); HRMS (ESI) m/z: calcd for $C_{15}H_{13}N_2O$ [M + H]$^+$ 237.1022, found 237.1025.

Notes and references

1. K. Upadhyaya, R. K. Thakur, S. K. Shukla and R. P. Tripathi, *J. Org. Chem.*, 2016, **81**, 5046.

2. J. K. Laha, K. S. S. Tummalapalli and K. P. Jethava, *Org. Biomol. Chem.*, 2016, **14**, 2473.

3. J. Fang and J. Zhou, *J. Org. Chem.*, 2011, **76**, 7730.

4. Y. Hu, L. Chen and B. Li, *RSC Adv.*, 2016, **6**, 65196.

5. W. Zhao, P. Liu and F. Li, *ChemCatChem*, 2016, **8**, 1523.

6. L. Yu, M. Wang , P. Li and L. Wang, *Appl. Organometal. Chem*. 2012, **26**, 576.

7. W. Xu and H, Fu, *J. Org. Chem.*, 2011, **76**, 3846.
